# Comparative Immunohistochemical Analysis of Craniopharyngioma and Ameloblastoma: Insights into Odontogenic Differentiation

**DOI:** 10.3390/diagnostics14202315

**Published:** 2024-10-17

**Authors:** Ban A. Salih, Bashar H. Abdullah

**Affiliations:** College of Dentistry, University of Baghdad, Baghdad 10071, Iraq; bashar.hamid@codental.uobaghdad.edu.iq

**Keywords:** craniopharyngioma, ameloblastoma, odontogenic differentiation, immunohistochemistry

## Abstract

Background and objectives: Histopathological similarities between craniopharyngioma (CP) and ameloblastoma (AB) have long been recognized, particularly the shared features of palisading columnar epithelium and stellate reticulum-like areas. This study aimed to investigate potential odontogenic differentiation in CP akin to AB using immunohistochemical odontogenic markers. Methods: We analyzed AMELX, ODAM, and CK19 expression in 44 cases (20 CP and 24 AB). Results: While AMELX and ODAM showed diffuse strong positive expression in both tumors with no significant statistical differences, CK19 expression was notably higher in CP. Conclusion: The markers AMELX and ODAM associated with odontogenic differentiation exhibited similar profiles in both tumors due to shared similar embryological origins. Notably, CK19, a biomarker of odontogenic epithelium, showed even higher expression, suggesting distinct pathways. These findings offer insights into tumor biology and may aid in diagnostic and therapeutic approaches.

## 1. Introduction

Craniopharyngiomas (CPs) are rare, histologically benign epithelial neoplasms that represent a significant challenge in neurosurgical practice. They constitute 1.2–4.6% of all primary intracranial tumors, where the incidence is estimated at 0.5–2 cases per million persons annually [1]. The first description of craniopharyngiomas was reported in 1857 by Zenker. This tumor was named craniopharyngioma by Cushing in 1932 and was widely established as a clinical entity [2]. CPs affect individuals across the lifespan, but their occurrence displays a distinct bimodal age distribution, peaking during childhood/early adolescence (5–15 years) and middle adulthood (44–56 years) [1]. While no clear gender predilection has been established, the anatomical location of these tumors, typically in the sellar/parasellar region, results in significant morbidity [2]. These tumors are generally diagnosed in patients who develop symptoms, including headaches or visual impairment (due to compression of the optic chiasm) and endocrine deficits from local mass effects on the hypothalamic–pituitary axis [2]. Management is further complicated by the fact that diagnosis of CP often has a long delay from symptom onset (up to 1–2 years) [2].

Craniopharyngiomas are rare in incidence, whereas ameloblastomas (ABs) were found to be the most common clinically significant odontogenic tumor; therefore, by itself, ameloblastoma equals all other odontogenic tumors except for limited cases of inside calcified composite [3,4]. Cusack first described ameloblastoma in 1827, which was later explained by Broca in 1868 [5,6]. Ameloblastoma was named adamantinoma by Baden in 1965 [7]. The term “ameloblastoma” was introduced in 1929 [7].

Although ABs can affect a wide age range, they are rarely observed in children younger than ten. The peak incidence is between the third and seventh decades of life, with no gender or ethnic predilection [3]. Most ABs (about 80%) arise from the mandible, especially in a molar/ramus location, and are manifested by an asymptomatic swelling [3].

Despite their distinct clinical presentations and sites of origin, CP and AB can pose diagnostic challenges due to their overlapping clinical and radiological features, particularly multilocular cystic formations [8]. The accurate differentiation of these tumors is crucial to guide appropriate treatment and management strategies.

Histopathologically, CPs are classified into two main subtypes: adamantinomatous (ACP) and papillary (PCP), with transitional or mixed forms also described [9]. The most prevalent subtype, ACP, typically presents as a large, multilobulated, solid cystic mass, often with calcifications and a characteristic “motor-oil”-like fluid content [9]. Microscopically, ACP shows islands of squamous epithelium in cords and trabeculae lined by palisading columnar cells [9]. Key diagnostic features include the presence of “stellate reticulum”-like areas, wet keratin, ghost cells, and reactive gliosis in surrounding brain tissue [9].

In contrast, PCP is almost exclusively observed in adults and tends to be a solid or mixed mass with viscous yellow cysts, rarely exhibiting calcifications [9]. Microscopically, PCP is composed of mature squamous epithelium forming pseudopapillae with an anastomosing fibrovascular stroma [9]. Notably, PCP lacks the peripheral palisading, stellate reticulum, and wet keratin characteristics of ACP [9].

Ameloblastomas also exhibit diverse histopathological patterns. Solid and multicystic ABs (which constitute approximately 75–86% of cases) are further subdivided into follicular, plexiform, acanthomatous, granular cell type, desmoplastic, and basal cell patterns [3]. The follicular type, which is the most common one, manifests leaf-like epithelial islands with peripheral columnar ameloblast-like cells and a central core of stellate reticulum-like cells [3]. The plexiform pattern consists of anastomosing cords or sheets of odontogenic epithelium bordered by ameloblast-like cells [3]. The other patterns are distinguished by varying degrees of squamous metaplasia, keratin formation, granular cell transformation, and desmoplasia [3].

The histopathological similarities between ACP and AB, particularly the shared features of palisading columnar epithelium and stellate reticulum-like areas, have long been recognized [8]. These resemblances are not mere coincidences but likely reflect their shared embryological origin from the oral ectoderm [8]. Both Rathke’s pouch, the presumed origin of CPs, and the odontogenic epithelium, the source of ABs, arise from this common precursor tissue [8,10,11]. The process of tooth development, initiated by the thickening and invagination of the oral epithelium into ectomesenchyme, mirrors the early stages of Rathke’s pouch development [10]. The shared developmental pathway provides a plausible explanation for the histological convergence of these tumors, prompting further investigation into their molecular underpinnings.

This study focuses on the comparative immunohistochemical analysis of three key markers: amelogenin (AMELX), odontogenic ameloblast-associated protein (ODAM), and cytokeratin 19 (CK19). These markers have been selected based on their well-established roles in tooth development and their potential relevance to the biology of CP and AB.

AMELX, a major enamel matrix protein, is crucial for enamel’s structural organization and biomineralization [12,13]. It is expressed by ameloblast in the secretory stage [14]. Low-molecular-weight enamel matrix protein has been consistently shown in the enamel organ’s inner enamel epithelium, stratum intermedium, and stellate reticulum [15]. While previously thought to be exclusive to enamel formation, AMELX expression has now been identified in other tissues, including bone and cartilage, suggesting a broader role in tissue development [13].

ODAM, another enamel protein, is vital in enamel maturation and mineralization [16]. Its expression has been detected in various epithelial neoplasms, including odontogenic tumors and cancers of the lung, breast, and stomach, suggesting a potential role in tumorigenesis [17]. Furthermore, ODAM has been proposed as a potential prognostic biomarker and therapeutic target for certain cancers [17].

CK19, a cytokeratin expressed in simple epithelia and various ductal tissues, is recognized as an essential biomarker of the odontogenic epithelium [18,19,20]. Its expression has been observed in several odontogenic tumors, including AB, highlighting its relevance to the study of these lesions [21,22]. Furthermore, CK19 was expressed in craniopharyngioma [9].

By comparing the expression profiles of AMELX, ODAM, and CK19 in CP and AB, this study aims to shed light on these tumors’ potential shared embryological origins and divergent pathological mechanisms. The findings could contribute to a better understanding of tumor biology, enhance diagnostic accuracy, and open avenues for targeted therapies in the future.

## 2. Materials and Methods

**Figure 1:**
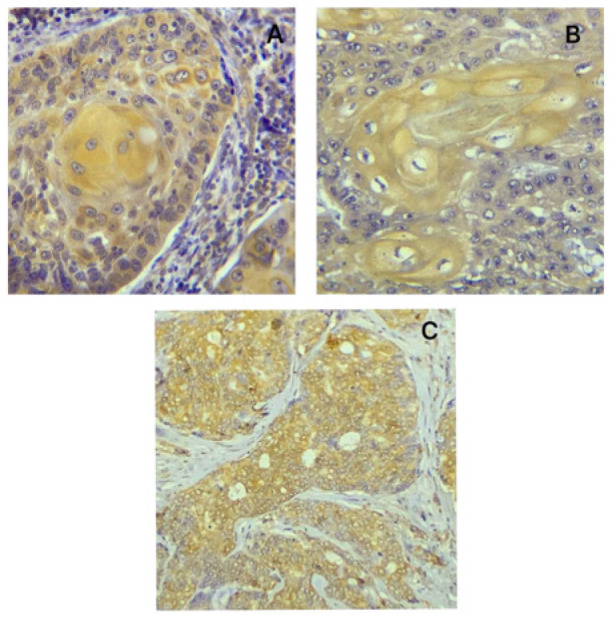
(**A**) Positive ODAM expression in the cytoplasm of the epithelioid cells and in the ghost cells of calcifying epithelial (Pindborg) odontogenic tumor that was used as a positive control for ODAM in the study (Immunohistochemistry, 400×). (**B**) Positive CK19 expression in the cytoplasm of the epithelial cells of the squamous cell carcinoma that was used as a positive control for CK19 in this study (Immunohistochemistry, 400×). (**C**) Breast carcinoma tissue used as a positive control for amelogenin in this study, with a positive cytoplasmic expression of amelogenin (Immunohistochemistry, 400×).

This study comprised 44 formalin-fixed tissue blocks, including 20 cases of craniopharyngioma and 24 cases of ameloblastoma. The primary antibodies used in this study included anti-amelogenin (AMELX, MyBioSource, San Diego, CA, USA), anti-ODAM (Abbexa, Houston, TX, USA), and anti-CK19 (MyBioSource). All the primary antibodies were polyclonal. Positive tissue controls were included in each run following manufacturer data sheets (Figure 1). Negative tissue controls were prepared by adding phosphate-buffered saline (PBS) instead of primary antibodies to the slides. The immunohistochemical procedure was conducted according to the instructions provided in the data sheets of the Abcam secondary antibody detection kit (ab80436-EXPOSE Rabbit and Mouse Specific HRP/DAB Kit, Abcam, Cambridge, UK). The detection system kit and the primary antibodies were put in the refrigerator at 4 °C. Each solution was allowed to reach room temperature before each step. 

All tissue specimens (cases and control) were presumably fixed in 10% formalin before being processed into paraffin blocks, cut into 4-micron thick sections, and placed on slides with a positive charge. The slides were incubated at 60 °C for two hours in a hot air oven. Then, rehydration was performed by immersing the slides twice in xylene (5 min each), followed by a serial of ethanol dilutions (100%, 100%, 95%, 75%, 50%) for 3 min each and then distilled water. The retrieving procedure involved pretreatment with heat-mediated antigen retrieving solution (citrate buffer pH 6.0). After heating the water-filled water bath and the Kaplan jars with the retrieval solution to 90–95 °C, the slides were placed into the jars and cooked for 20 min at that same temperature. After that, the slides were cooled at room temperature for an additional 25 min. A protein block was added after applying a peroxidase block and incubating for 10 min at 37 °C to inhibit endogenous peroxidase activity. Next, each slide received at least 100 μL of diluted primary antibody and was incubated for an hour, and they were then put in the refrigerator at 4 °C overnight. The next day, secondary antibody and HRP conjugate were applied. The slides were then incubated in a humid chamber for 15 min in the incubator, followed by two rinses with PBS for 5 min each. Chromogen and DAB (Diamino Benzidine) substrates were applied to each slide, and the slides were placed away from light for 10 min. Hematoxylin counterstaining was performed by rinsing the slides for 30 s and then washing the slides with running water. Dehydration was performed to remove the water contained in the tissue by immersing the slides in a series of ethanol dilutions (50%, 75%, 95%, 100%- 100%), followed by immersion twice in xylene (5 min each) and covering with DPX mounting media.

Evaluation of the immunohistochemistry involved examining positive slides by counting the positive cells of ameloblasts and stellate reticulum in 5 high-power fields (400×). For amelogenin staining, the assessment was categorized as follows: “−” indicated no positive cells; “+”, less than 10%; “++”, 10–50%; “+++”, over 50% positivity [10]. Similar to this, the proportion of positive cells was used to determine the score for ODAM staining, which ranged from “−” (0–4%) to “+++” (>75%) [23]. According to the following criteria, CK19 staining was assessed: “−” indicated fewer than 10% positive cells; “+”, 10% to 50% positive cells; “2+”, more than 50% positive cells [24].

Given the nature of our data and the study’s sample size, we carefully chose acceptable statistical tests for comparing categorical data. The primary consideration was the distribution and expected frequency of data points within each category of our contingency tables.

The Chi-squared test is frequently utilized with larger sample sizes and when the expected frequencies in each category of the contingency table exceed 5. This condition ensures that the approximations used to calculate the *p*-values are valid.

However, in our study, several categories had expected frequencies of less than five due to the small sample size in each group (20 cases of craniopharyngioma and 24 cases of ameloblastoma). This circumstance often leads to a significant chance of type I errors (incorrectly rejecting the null hypothesis) when employing the Chi-squared test.

As a result, we chose Fisher’s exact test, specifically developed for small sample sizes and robust to data distribution within the contingency table. Unlike the Chi-squared test, Fisher’s exact test does not rely on large sample assumptions and is more appropriate for our study, where the cell counts are low. This test yields an exact *p*-value based on the hypergeometric distribution, making it a better choice for our comparative investigation of immunohistochemical marker expression in craniopharyngioma and ameloblastoma. Using Fisher’s exact test ensures the accuracy of our findings, reducing the risk of statistical error caused by small, uneven sample numbers across the compared groups.

By employing Fisher’s exact test, we adhere to statistical best practices, providing a strong analytical framework that supports our findings’ validity and thereby improves our study’s scientific rigor.

## 3. Results

In total, 44 cases were examined, comprising 20 cases of craniopharyngioma and 24 cases of ameloblastoma. Among the craniopharyngioma cases, 18 were of the adamantinomatous subtype (nine cystic, eight follicular, and one plexiform-like) while 2 were papillary. Fourteen cases of the adamantinomatous type exhibited ghost cells. Regarding ameloblastoma, 7 cases were cystic, 5 were plexiform, and 12 were follicular.

The immunohistochemical analysis of AMELX, ODAM, and CK19 across the 44 cases revealed distinct expression patterns between craniopharyngioma and ameloblastoma. The results are summarized in Table 1, which includes frequencies, percentages, and confidence intervals for each marker expression category. However, the expression patterns varied considerably among the cases. Some cases exhibited only ameloblast or stellate reticulum expression, while others showed both.

**Figure 2:**
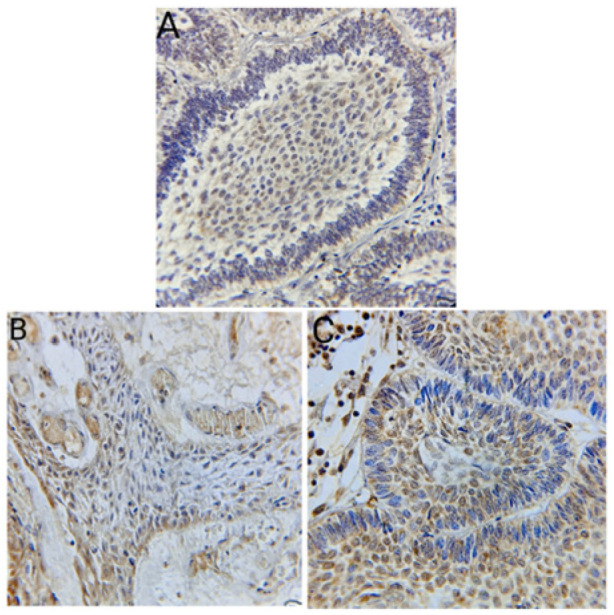
(**A**) Follicular ameloblastoma with cytoplasmic expression of amelogenin antibody in the stellate reticulum and peripheral layer resembling ameloblast (Immunohistochemistry, 400×). (**B**) Papillary craniopharyngioma positive cytoplasmic expression of amelogenin antibody in the squamous epithelial cells (Immunohistochemistry, 400×). (**C**) Adamantinomatous craniopharyngioma with a cytoplasmic expression of amelogenin antibody in both stellate-like area and peripheral ameloblast (Immunohistochemistry, 400×).

Regarding amelogenin expression, there were no significant differences between craniopharyngioma and ameloblastoma (*p* = 0.320) (Figure 2A–C). Most cases of both diagnoses demonstrated strong amelogenin positivity (+3). Notably, 90% of adamantinomatous craniopharyngioma cases with ghost cells showed strong amelogenin expression in these cells.

**Figure 3:**
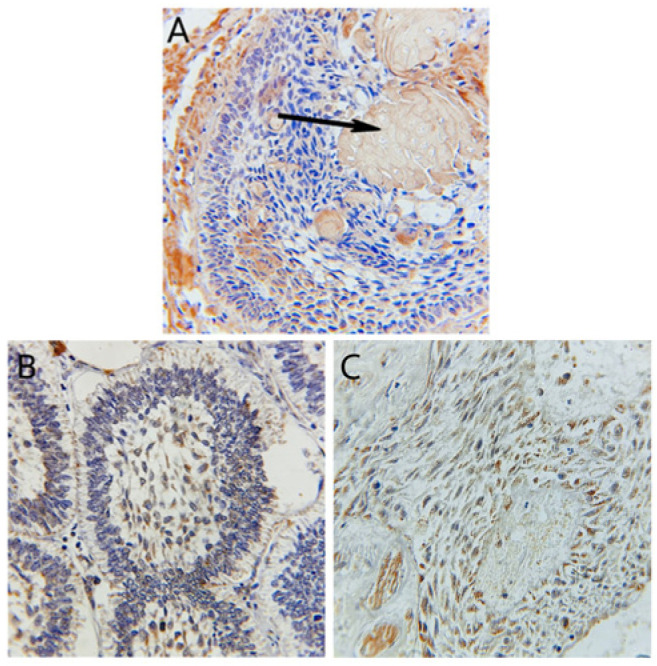
(**A**) Adamantinomatous craniopharyngioma with cytoplasmic expression of ODAM antibody in both ameloblast and stellate-like area, with positive expression of ODAM in the ghost cells (black arrow) (Immunohistochemistry, 400×). (**B**) Follicular ameloblastoma with cytoplasmic expression of ODAM antibody in both stellate reticulum and ameloblast (Immunohistochemistry, 400×). (**C**) Papillary craniopharyngioma with membranous expression of ODAM antibody in the squamous epithelial cells (Immunohistochemistry, 400×).

ODAM expression also did not significantly differ between craniopharyngioma and ameloblastoma (*p* = 0.209), with the majority of cases showing strong positive expression (+3) (Figure 3A–C). Notably, ODAM expression was observed in both ameloblast-like cells and stellate reticulum in 61% of cases. In comparison, it was observed solely in stellate reticulum in 30% of cases and ameloblast-like cells in four cases. Ghost cells in adamantinomatous craniopharyngioma cases also exhibited strong ODAM expression.

**Figure 4:**
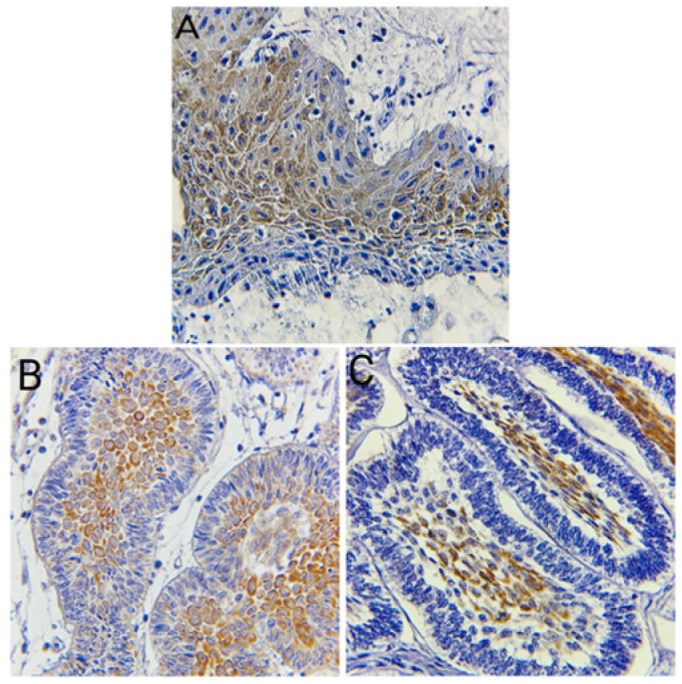
(**A**) Papillary craniopharyngioma with cytoplasmic expression of CK19 antibody in the squamous epithelial cells (Immunohistochemistry, 400×). (**B**) Adamantinomatous craniopharyngioma with membranous expression of CK19 antibody in the stellate-like area (Immunohistochemistry, 400×). (**C**) Follicular ameloblastoma with cytoplasmic expression of CK19 antibody in the stellate reticulum-like area (Immunohistochemistry, 400×).

The expression of CK19 was significantly higher in craniopharyngioma compared to ameloblastoma (*p* = 0.008), with most craniopharyngioma cases showing strong positivity (+2) (Figure 4A–C). The effect size calculated (phi coefficient = 0.42) indicates a moderate association. Notably, strong CK19 expression was observed in the stellate reticulum, with 54.5% of cases showing expression solely in the stellate reticulum and 38.5% showing expression in both ameloblast-like cells and stellate reticulum. Only 7% of cases exhibited CK19 expression solely in ameloblast-like cells. Ghost cells also demonstrated strong CK19 expression.

**Figure 5:**
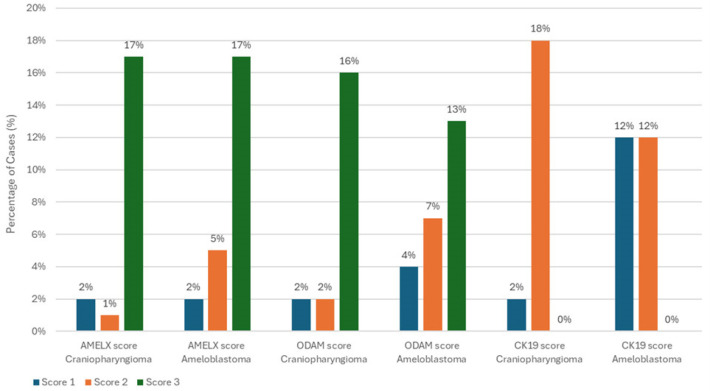
Comparison of AMELX, ODAM, and CK19 expression levels between craniopharyngioma and ameloblastoma.

Figure 2, Figure 3 and Figure 4 illustrate the expression patterns of AMELX, ODAM, and CK19, respectively, visually comparing the two types of tumors. Figure 5 includes bar graphs that represent the percentage of cases showing each marker’s expression level, enhancing the interpretability of the results. These figures visually demonstrate the differences and similarities in marker expression between craniopharyngioma and ameloblastoma, highlighting the unique immunohistochemical profiles observed.

In summary, while amelogenin and ODAM expression did not significantly differ between craniopharyngioma and ameloblastoma, CK19 expression was significantly higher in craniopharyngioma. These findings underscore the varied immunohistochemical profiles between the two tumor types and may provide insights into their underlying molecular characteristics and developmental pathways.

## 4. Discussion

This study investigated the immunohistochemical expression of amelogenin (AMELX), odontogenic ameloblast-associated protein (ODAM), and cytokeratin 19 (CK19) in craniopharyngioma and ameloblastoma, aiming to shed light on their potential shared embryological origins and divergent pathological mechanisms. Our findings revealed similarities in AMELX and ODAM expression between these two tumor types, supporting the hypothesis of a shared embryological origin from the oral ectoderm. This aligns with previous research indicating the role of amelogenin, an enamel protein synthesized by ameloblasts [10], as an indication of the development of epithelial cells in odontogenic lesions [25] and a predictor of histological behavior. Amelogenin’s utility as a definitive marker for identifying and differentiating odontogenic lesions from other epithelial lesions in the oral and maxillofacial regions has been well-established [15].

Similarly, ODAM, a developmental antigen crucial for tooth maturation, plays a role in the pathogenesis of various odontogenic and epithelial neoplasms [17]. Its expression in ameloblast differentiation and enamel mineralization [19] further supports the odontogenic differentiation of ameloblastoma. 

The similarities in AMELX and ODAM expression between these tumors support the hypothesis of a shared embryological origin. Craniopharyngiomas are thought to arise from epithelial remnants of Rathke’s pouch [8], while ameloblastomas originate from oral epithelium and odontogenic cells [7]. Both Rathke’s pouch and odontogenic epithelium are derived from the stomodeal ectoderm, a process involving the thickening and invagination of the oral epithelium into ectomesenchyme [10]. This common embryological origin may explain the shared ODAM and AMELX expression between these intracranial and intraosseous tumors.

However, despite these shared characteristics, our study revealed a key difference in the expression of CK19. The significantly higher expression of CK19 in craniopharyngioma compared to ameloblastoma suggests a potentially distinct pathway in its odontogenic differentiation. This finding is particularly intriguing considering the role of CK19 as a marker of odontogenic epithelium, as previously established [24,26]. CK19 is known to be upregulated during the conversion of inner enamel epithelium to ameloblasts and is considered a stem cell marker for understanding the pathogenesis of odontogenic cysts and tumors [26]. The expression of CK19 in craniopharyngioma has also been reported by Campanini et al. [9]. Reduced enamel epithelium with overexpressed CK19 indicates the immaturity of the tumor cell lineage, may interfere with terminal differentiation, and may show the capacity of cells to proliferate [26]. These findings, along with the hypothesis of the shared embryological origin of these two tumors, further support the odontogenic differentiation of craniopharyngioma.

Further, the significantly higher CK19 expression in craniopharyngioma holds exciting implications for the diagnosis and prognosis of these tumors, since CK19 was considered a strong epithelial tumor marker used to diagnose and evaluate the prognosis of various tumors of epithelial origin [27]. Additionally, CK19 serves as a useful research tool in the prognosis, diagnosis, and management of both tumors owing to its overexpression in several tumors [28]. In other studies, associations between reduced CK19 expression and some unfavorable phenotypic tumor features and poor patient prognosis were discovered [29]. 

The differential expression of CK19 may reflect variations in the underlying signaling pathways driving tumor development in craniopharyngioma and ameloblastoma. While both tumors exhibit dysregulation of the Wnt signaling pathway, a key player in tooth development [30], the specific downstream effectors and their influence on CK19 expression may differ. This divergence could be attributed to the distinct microenvironments and cellular contexts in which these tumors arise. Craniopharyngiomas develop in the sellar/suprasellar region, which is surrounded by pituitary tissue and influenced by hormonal factors. At the same time, ameloblastomas arise within the jawbones, interacting with a different set of surrounding cells and signaling molecules. These distinct environments could lead to variations in signaling pathways’ activation and downstream effects, ultimately impacting CK19 expression.

While our study provides valuable insights, it is important to acknowledge its limitations. The retrospective design and relatively small sample size limit the ability to control for potential confounding factors and generalize the findings to all populations. The inherent heterogeneity of craniopharyngiomas and ameloblastomas, with their diverse histological subtypes, might also have influenced the observed marker expression patterns. Technical variations inherent to immunohistochemical staining, such as differences in tissue fixation, processing, and antibody incubation times, could also have contributed to variability in the results.

Future studies with larger, well-characterized cohorts are warranted to validate these findings. These studies should include detailed analyses of different histological subtypes and correlate marker expression with clinical outcomes, such as tumor recurrence and overall survival. Further investigation into the specific molecular mechanisms underlying the differential expression of CK19 and its role in tumor development is also crucial. This could involve examining the expression and activity of downstream effectors of the Wnt signaling pathway and exploring potential interactions with other signaling pathways.

## 5. Conclusions

In conclusion, our study demonstrates similarities in AMELX and ODAM expression between craniopharyngioma and ameloblastoma, supporting their shared embryological origins. However, the significantly higher expression of CK19 in craniopharyngioma suggests a distinct pathway in its odontogenic differentiation. This finding highlights CK19 as a potential diagnostic marker and therapeutic target for craniopharyngioma. By advancing our understanding of these tumors’ immunohistochemical profiles, we contribute to the broader efforts in refining diagnostic accuracy and exploring new treatment options.

## Figures and Tables

**Table 1:** Comparison of AMELX, ODAM, and CK19 expression between craniopharyngioma and ameloblastoma using Fisher’s exact test.

Marker	Diagnosis	Score 1 (*n*, %, 95% CI)	Score 2 (*n*, %, 95% CI)	Score 3 (*n*, %, 95% CI)	*p*-Value
AMELX	Craniopharyngioma	2 (9.1%, 2.3–29.0%)	1 (4.5%, 0.8–20.4%)	17 (77.3%, 58.9–89.6%)	0.32
	Ameloblastoma	2 (8.3%, 2.1–27.0%)	5 (20.8%, 9.1–41.7%)	17 (70.8%, 52.8–84.7%)	
ODAM	Craniopharyngioma	2 (9.1%, 2.3–29.0%)	2 (9.1%, 2.3–29.0%)	16 (72.7%, 54.3–85.4%)	0.209
	Ameloblastoma	4 (16.7%, 6.6–36.6%)	7 (29.2%, 15.3–48.7%)	13 (54.2%, 34.2–73.0%)	
CK19	Craniopharyngioma	2 (9.1%, 2.3–29.0%)	18 (81.8%, 63.6–92.3%)	0 (0%, 0–15.4%)	0.008
	Ameloblastoma	12 (50.0%, 30.7–69.3%)	12 (50.0%, 30.7–69.3%)	0 (0%, 0–15.4%)	

Note: Confidence intervals (CI) for percentages are based on the Wilson score method. AMELX—amelogenin; ODAM—odontogenic ameloblast-associated protein; CK19—cytokeratin19.

## Data Availability

The data supporting this study’s findings are available from the corresponding author upon reasonable request.
